# Leiomyomatosis peritonealis disseminata with low-grade malignant change: A case report

**DOI:** 10.1097/MD.0000000000030528

**Published:** 2022-09-09

**Authors:** Zhongxue Ye, Lu Chen

**Affiliations:** a Department of Gynecology, Hwa Mei Hospital of University of Chinese Academy of Sciences, Ningbo, China; b Department of Gynecologic Oncology, Cancer Hospital of University of Chinese Academy of Sciences, China; c Department of Gynecologic Oncology, Zhejiang Cancer Hospital, Hangzhou, China; d Institute of Cancer Research and Basic Medical Sciences, Chinese Academy of Sciences, Hangzhou, China.

**Keywords:** case report, laparoscopic myomectomy, leiomyomatosis peritonealis disseminata, low potential malignant

## Abstract

Leiomyomatosis peritonealis disseminata (LPD) is a rare disease characterized by multiple leiomyomas spread all over the peritoneal cavity. It is commonly described as benign in women of reproductive age. Malignant LPD is much rarer.

**Methods:**

We present a case of LPD with low potential malignant change in a 43-year-old female, who felt a lump in her abdomen after laparoscopic myomectomy 10 years ago and laparoscopic hysterectomy 8 years ago. The patient underwent exploratory laparotomy and salpingectomy, greater omentectomy, and pelvic and abdominal mass resection were performed during the surgery. The pathological findings revealed LPD with low potential malignant change, with strong expression of estrogen receptor and progesterone receptor. The patient refused oophorectomy and chose gonadotropin-releasing hormone agonists injection postoperatively.

**Results:**

No recurrence was found during the follow-up to date.

**Conclusion:**

Surgery is the main treatment for LPD, and endocrine therapy is another choice. Although it is reported mostly benign, we need to be alert to the possibility of malignancy.

## 1. Introduction

Leiomyomatosis peritonealis disseminata (LPD) is a rare disease characterized by multiple leiomyomas scattered in the peritoneum. It was first reported in 1952 by Willson and Peale^[1]^ and named by Taubert et al^[2]^ in 1965. Up to now, >200 cases have been reported. LPD usually occurs in reproductive-aged women. Occasionally, LPD has been reported in postmenopausal women and men.^[3]^ The exact mechanism of LPD is still unclear, although there have been several related theories, such as hormones, stem cell metaplasia, and genetic or iatrogenic implantation after myoma fragmentation during laparoscopic surgery.^[4–7]^ Continuous hormonal stimulation such as ovarian tumors that secrete estrogen or taking oral sex hormones may also be a risk factor.^[8]^ Most patients are asymptomatic and are discovered by accident. Some patients presented as abdominal mass, abdominal pain, or intestinal obstruction. Most cases reported in the literature were benign; however, there were still a few reported as malignant.^[9]^ The majority of malignant cases were described concurrence of LPD and leiomyosarcoma, or malignant transformation to leiomyosarcoma from LPD.^[10–12]^ Because of its rarity, there is a lack of consensus on the standard treatment for LDP and the prior treatment is surgery lies on the case reports.^[13]^ Here, we reported a case of LPD with low potential malignant change from Zhejiang Cancer Hospital in September 2020. By sharing this case and reviewing associated literature, we hope to further enhance the understanding of LDP for medical workers, which may benefit for future diagnosis and treatment.

## 2. Case report

**Figure 1.**
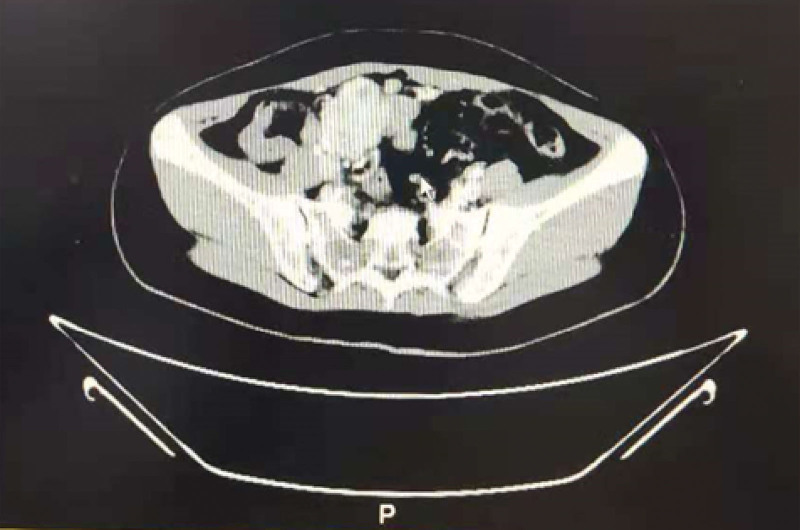
Enhanced computed tomography scan revealed irregular blood-supplying masses in the abdominal and pelvic cavity.

A 43-year-old female was admitted for treatment of pelvic and abdominal masses. The patient gave written informed consent to publish this case report. She had a history of 4 pregnancies and 1 birth. Ten years ago, she underwent laparoscopic myomectomy. The pathological report suggested cellular leiomyoma. Half a year later, she was diagnosed with recurrent uterine fibroids during follow-up. Eight years ago, she underwent laparoscopic hysterectomy. Uterine fibroid of about 4 cm was found intraoperatively. The pathological report still suggested cellular leiomyoma. Thereafter, she did not receive any treatment and did not follow up regularly. In August 2020, she felt a lump in her abdomen and went to the hospital. Physical examination found several irregular masses in the pelvic and abdominal cavity with no tenderness and poor movement. The larger one was located in the pelvic cavity, about 7 × 6 cm. Transvaginal ultrasound showed multiple hypoechoic nodules with peritoneal effusion in the abdominal cavity, the largest of which was about 8 × 5 cm. Contrast-enhanced computed tomography revealed multiple blood-supplying nodules (Fig. 1) in the abdominal and pelvic cavity, with a small amount of peritoneal effusion, indicating metastatic tumor to be disposed. Laboratory tests showed a CA125 level of 42.1 U/mL. Following preoperative evaluation, exploratory laparotomy was scheduled.

**Figure 2.**
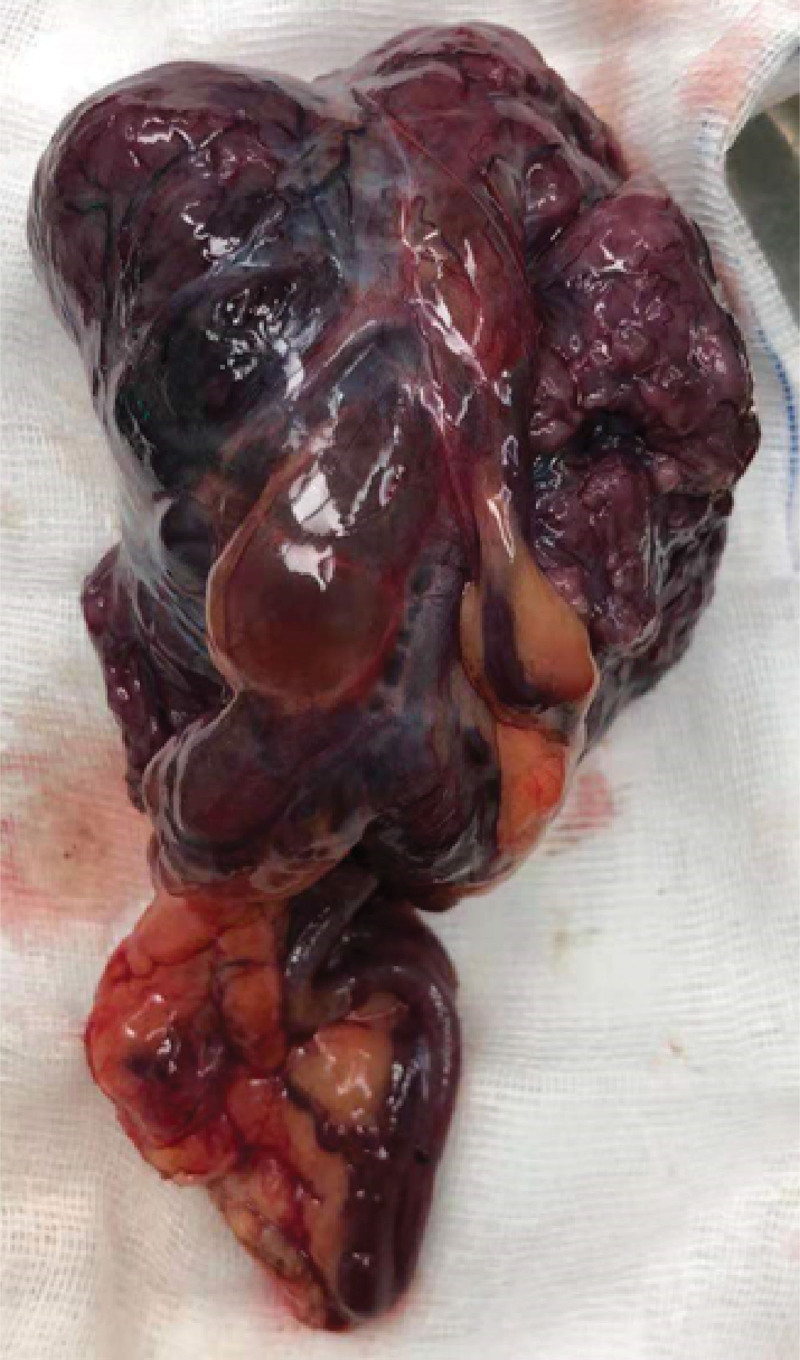
The biggest mass located in the right pelvic cavity, about 7 × 6 cm.

**Figure 3.**
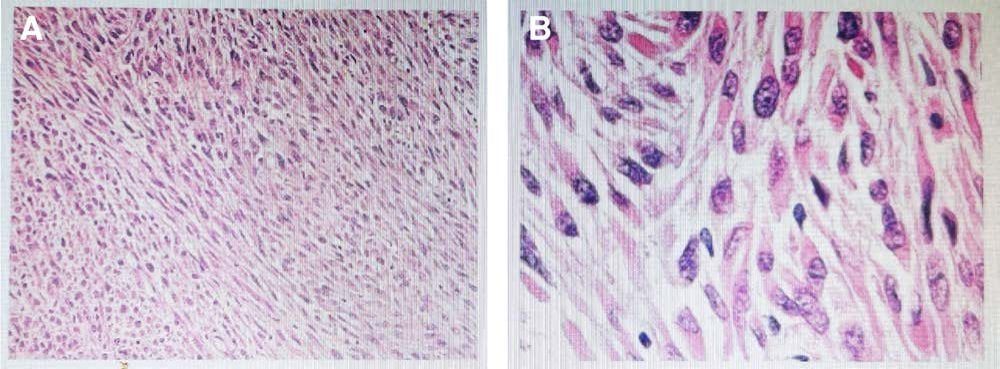
The pathological analysis showed the smooth muscle tumor with active cell growth and focal necrosis (H&E, A×100, B×400).

During the operation, several nodules, about 0.1 to 0.5 cm in size, were observed near the original puncture hole. Multiple nodules, about 0.1 to 1.0 cm in size, were detected in the greater omentum. Blood vessels on the surface of the greater omentum were obviously thickened; there were 2 lobulated masses on the mesentery of the sigmoid colon with abundant blood vessels, about 5 and 3 cm in diameter, respectively. A lobulated mass on the left side of the bladder was observed about 6 × 5 cm, adhered between the pelvic wall and bladder. Another mass detected in the left rectal fossa was about 3 × 3 cm. The biggest mass was about 7 × 6 cm, located in the right pelvic cavity (Fig. 2). The mass is lobulated and dark red with rich thickened blood vessels. Several miliary nodes about 0.1 to 0.3 cm on the bilateral fallopian tubes were found and multiple nodules about 0.1 to 2.0 cm were observed on the surface of the small mesentery, mesocolon, bilateral paracolic sulcus, bilateral abdominal, and pelvic peritoneum. The biggest mass and the sigmoid mesangial mass were removed and the intraoperative pathological detection of them showed leiomyoma, part of cells in rich. Finally, Salpingectomy, greater omentectomy, and pelvic and abdominal mass resection were performed. The histopathologic analysis showed that the masses were smooth muscle tumor with active cell growth and focal necrosis, with 1 to 5 mitotic figures per 25 high-power fields, which tended to be low-grade malignant leiomyosarcoma (Fig. 3). The immunohistochemistry suggested high expression of estrogen receptor (ER) (+++, 90%) and progesterone receptor (PR) (+++, 95%). The patient requested preservation of the ovaries and chose gonadotropin-releasing hormone agonists (GnRHa) injection monthly postoperatively. There were no obvious adverse events during the treatment. No recurrence was found during the follow-up to date.

## 3. Discussion

LPD is a rare disease, which was first reported by Willson and Peale^[1]^ in 1952. To date, >200 cases have been founded worldwide. Its etiology is still unclear. Hormone hypothesis is the original and classic theory about the onset of LPD, which suggests that hormones are closely related to the occurrence and development of LPD. The hypothesis was demonstrated in patients with pregnancy, or taking contraceptive, hormone replacement therapy or tamoxifen therapy, confirmed with estrogen-secreting tumors, or ovarian stimulation.^[14–16]^ In addition, some researchers have found that lowering estrogen levels with GnRHa or aromatase inhibitors can make LPD tumor nodules regress.^[16]^ The findings of ER and PR in LPD further proved the important role of hormones in the pathogenesis of LPD.^[17]^ However, hormones are not the only cause of LPD. There were also cases reported about LPD found in menopausal women, and men, who have never or rarely exposed to hormones.^[3]^ Thus, some scholars put forward that LPD may originate from peritoneal mesenchymal stem cells. In recent years, there have been increasing reports of LPD after laparoscopic surgery.^[18]^ The common feature of these patients is the use of electric excision for uterine morcellation or tumor morcellation. In addition, Momtahan et al^[19]^ found that LPD tumor nodules grew on the scar of laparoscopic puncture foramen after previous myomectomy, and the patients did not receive any hormone therapy. All these have prompted scholars to consider the causes of LPD: whether it is iatrogenic? In the process of tumor morcellation, small fibroid tissues and cells were shed and left in the abdominal cavity. Under the stimulation of various factors, they proliferated and formed new blood vessels with surrounding tissues, and grew gradually until developing to LPD, especially for women who are genetically susceptible. This theory has been accepted by more and more researchers. In our report, the patient did not receive any hormone therapy before but had undergone laparoscopic uterine fibroid morcellation. Therefore, laparoscopic uterine fibroid morcellation may be an important factor for secondary disseminating of fibroids.

LPD is often asymptomatic or presents with nonspecific symptoms, such as abdominal discomfort, abdominal pain or pelvic pain, abdominal mass, etc. A few cases have reported intestinal obstruction and omentum greater torsion caused by LPD.^[20,21]^ LPD can be radiologically manifested as abdominal spread similarly to malignant tumors. It is difficult to distinguish small malignant peritoneal implants from LPD nodules. Nodules of LPD tumor may present as localized, multiple, and fixed nodules on the peritoneal and mesenteric surfaces, but without ascites or other signs of malignancy. Thus, preoperative diagnosis of LPD is difficult.

LPD is mostly benign, but there were a few cases of malignant changes have been reported.^[10,11,22]^ Our case reported a low-grade malignant leiomyosarcoma by the final immunohistochemical pathology, accompanied by estrogen and PR expression. The risk factors for malignant transformation of LPD are still unclear.

There are no clinical guidelines for the treatment of LPD. Most researchers believe that surgical treatment is the first choice. For childless LPD patients, if surgical treatment is needed, it should be performed after the cessation of hormone therapy, and the surgical scope should include resection of the tumor and omentum greater. For LPD patients who have completed childbirth, hysterectomy, bilateral adnexectomy, greater omentum resection, and tumor resection may be a better choice. Of course, relapse may occur even after surgical treatment in patients of LPD. On the other hand, since most LPD occurs in women of childbearing age, conservative treatment should also be considered. First, endogenous or exogenous hormonal stimulation must be discontinued. GnRHa, aromatase inhibitors, progesterone, and ulipristal acetate may be a choice for treatment.^[23,24]^ Combined with our case, the patient was diagnosed with low-grade malignant leiomyosarcoma and she refused another operation to remove her ovaries. Thus, GnRHa was chosen for her further treatment. In conclusion, the patients’ age, symptoms, fertility requirements, previous treatment, and other factors should be taken into full consideration, and personalized treatment should be implemented with respect to the patients’ wishes.

## 4. Conclusion

To sum up, LPD is a very rare disorder. Here, we reported a reproductive-aged woman diagnosed as LPD with low potential malignant change. Based on a history review, we believed that laparoscopic myomectomy and laparoscopic hysterectomy might be risk factors for her. In view of the theory of iatrogenic pathogeny, the residual fibroid fragments should be avoided during uterine fibroid morcellation. Preoperative examination and rapid intraoperative pathological detection may have difficulty in accurate diagnosis. The final nature of the mass depends on final pathology and, if necessary, combined with immunohistochemistry result. Surgery is the main treatment for the disease. Endocrine therapy may be another choice for treatment, and it can be used as a complementary option sometimes. In addition, LPD still needs to be continuously explored in order to improve the level of diagnosis and treatment of LPD.

## Author contributions

Conceptualization: Zhongxue Ye and Lu Chen.

Data curation: Zhongxue Ye.

Formal analysis: Zhongxue Ye.

Supervision: Lu Chen.

Writing–original draft: Zhongxue Ye.

Writing–review & editing: Lu Chen.
